# Effectiveness of scheduled postoperative intravenous acetaminophen for colon cancer surgery pain

**DOI:** 10.1186/s40780-018-0115-1

**Published:** 2018-07-06

**Authors:** Eiji Horita, Yuji Takahashi, Kojiro Takashima, Kenichiro Saito, Yoshihiro Takashima, Yoshinori Munemoto

**Affiliations:** 1 Department of Pharmacy, Fukui-ken Saiseikai Hospital, 7-1, Funabashi, Wadanaka-cho, Fukui 918-8503 Japan; 2 Department of Surgery, Fukui-ken Saiseikai Hospital, 7-1, Funabashi, Wadanaka-cho, Fukui 918-8503 Japan

**Keywords:** Colon Cancer, Postoperative pain, Acetaminophen, Alanine aminotransferase

## Abstract

**Background:**

Clinical cases are often observed when patients are in need of repeated use of analgesic infusion to manage pain after colon cancer surgery. This paper investigates analgesic frequency as well as safety of postoperative intravenous (IV) acetaminophen in colon cancer surgery where epidural anesthesia is used.

**Methods:**

Among patients who received epidural anesthesia during colon cancer surgery, one group of twenty eight (28) patients received acetaminophen while another group of patients (30) did not receive it. The groups were analyzed from the surgery day to two days after for the postoperative occurrence and frequency of liver dysfunction in relation to analgesic usage.

**Results:**

The patient group with acetaminophen infusion significantly reduced the amount of analgesic medication compared to the group without the treatment (*p* = 0.008). Furthermore there was a significantly larger number of patients in the group receiving acetaminophen treatment with the baseline increase of alanine aminotransferase (*p* = 0.043). In most of the cases, however, the rate of the increase is mild and the patients did not need medication and subsequently recovered quickly.

**Conclusions:**

Scheduled IV infusion of acetaminophen after colon cancer surgey is concluded an effective method of pain control and alleviation of postoperative discomfort from the surgery day to two days after the surgery.

## Background

Any medical surgery provokes patient anxiety. Patients who have undergone digestive system surgery, in particular, have been reported to experience severe postoperative pain [1]. We witnessed many cases where additional analgesics such as pentazocine or non-steroidal anti-inflammatory drugs (NSAIDs) reduced postoperative pain after colon cancer surgery. The American Anesthesiology Association recommends a scheduled infusion of NSAIDs and acetaminophen as multi-modal analgesics, unless their use is prohibited in the guidelines for managing acute postoperative pain [2]. It has been reported that the scheduled concomitant infusion of acetaminophen after laparoscopic surgery alleviated postoperative pain and subsequently enhanced patient satisfaction [3].

Furthermore, according to the report by Zafar et al., a concomitant infusion of acetaminophen after laparoscopic colon removal surgery favourably contributed to shortening the hours required before resuming a normal diet, as well as reduced the number of days of hospitalization [4]. Encouraged by these results, we examined the frequency and safety of administration of postoperative analgesics and how the scheduled concomitant IV infusion of acetaminophen affected the colon cancer surgery in the cases when epidural anaesthesia was administered.

## Methods

### Subjects

From mid-February 2017, we began administering 1000 mg of postoperative IV acetaminophen every 6 hours from the day of surgery to the second day after the surgery when a patient might not have been able to take enough oral medication. Twenty-eight (28) colon cancer surgery patients who received epidural anaesthesia from March to May 2017 were grouped in an acetaminophen group (A).

The control group (C), on the other hand, consisted of thirty (30) colon cancer patients who received epidural anaesthesia for surgery from March to May 2016. Group C did not receive acetaminophen before the specified period.

### Research method

We conducted retrospective research by using electronic medical records.

To evaluate the patients’ background, we examined their age, sex, weight, surgical method (abdominal or laparoscopic), clinical stage, presence or absence of liver metastasis, and grades of aspartate aminotransferase (AST) and alanine aminotransferase (ALT).

We evaluated the AST and ALT grades, from G1 to G4, by using the Common Terminology Criteria for Adverse Events (CTCAE), v. 4.0. When the test value was within the standard range for the facility (AST < 40 U/L and ALT< 45 U/L), we evaluated it as G-0. In terms of the postoperative pain level, we first studied the frequency of non-opioid analgesic infusion use for pain management from the surgery day to 2 days after the surgery and compared the frequency of analgesic usage between Group A and Group C.

To evaluate the level of liver dysfunction, we used AST and ALT values.

We examined preoperative and postoperative levels on the 7th day. When the value increased more than one grade, we categorized it as a presence of side effects. For Group A only, we also evaluated the grade on the third day after operation.

### Statistical analysis

To evaluate statistical significance, we used Fisher’s exact probability test and the Wilcoxon rank-sum test using the Statistical Package for Social Science (SPSS) version 24.0 for Windows (SPSS, Inc., Chicago, IL, USA). A *P*-value of < 0.05 was considered significant.

## Results

### Characteristics of patients

There were no significant differences between Group A and Group C in terms of the patients’ age, sex, weight, surgical method (abdominal or laparoscopic), clinical stages, presence or absence of liver metastasis, and AST and ALT grade evaluation (Table 1).

**Table 1:** Patients’ background

	Group A (*n* = 28)	Group C (*n* = 30)	*p*
Patients’ age: mean, years	65.3 ± 2.5	68.3 ± 2.6	0.335^a^
Sex			1.000^b^
Male	15	17	
Female	13	13	
Body weight: mean, kg	59.0 ± 2.5	56.8 ± 2.5	0.796^a^
Surgical method			0.798^b^
Abdominal	16	16	
Laparoscopic	12	14	
Clinical stage			0.732^b^
I	8	10	
II	6	8	
IIIa	4	5	
IIIb	2	3	
IV	8	4	
Liver metastasis			0.726^b^
Presence	5	4	
Absence	23	26	
AST			0.344^b^
G0	25	29	
G1	3	1	
G2	0	0	
G3	0	0	
G4	0	0	
ALT			1.000^b^
G0	27	29	
G1	1	1	
G2	0	0	
G3	0	0	
G4	0	0	

^a^Used the Wilcoxon rank sun test for statistical analysis

^b^Used the Fisher exact probability test

### Postoperative pain evaluation

**Fig. 1:**
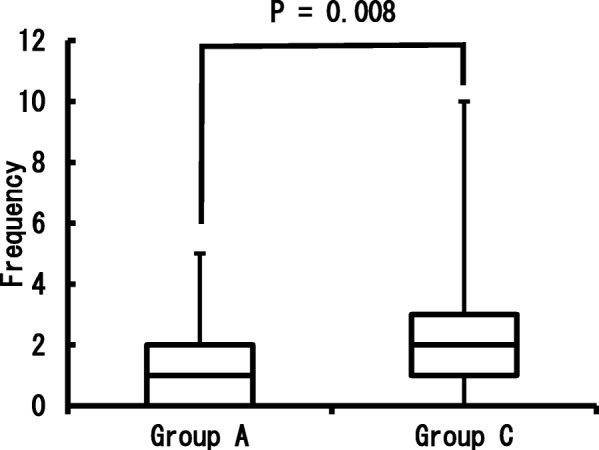
Frequency of the analgesics usage for postoperative pain.Comparison of the analgesics usage during the surgery day to 2 day after. Used the Wilcoxon rank sum test for the statistical analysis

Compared to Group C, which had an average use of non-opioid analgesics of 2.6 times and a median of 2 times, Group A had a significantly reduced use of non-opioid analgesics (average of 0.3 times and a median of 1 time) (*p* = 0.008) (Fig. 1).

### Safety

**Fig. 2:**
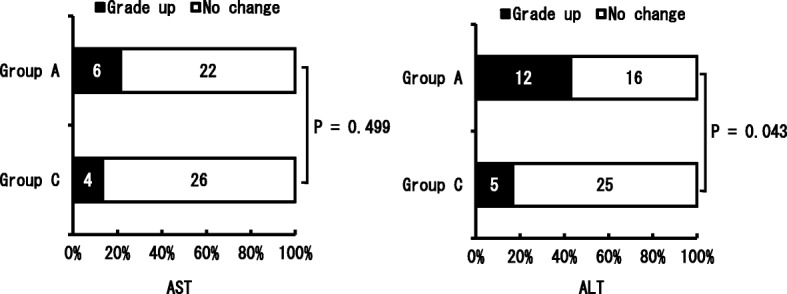
Comparison of the AST and ALT which was raised more than one grade up depending on the usage of Acetoaminophen. Used the Fisher’s exact probability test

**Fig. 3:**
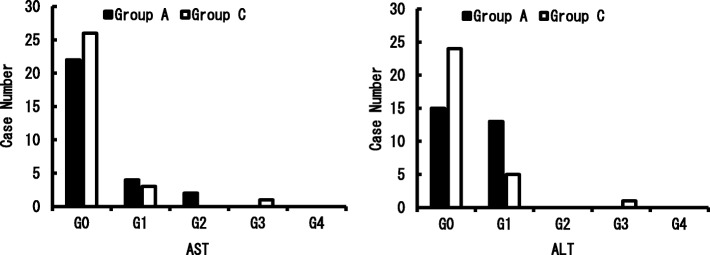
Postoperative Day7: Number of cases and grade of AST and ALT

**Fig. 4:**
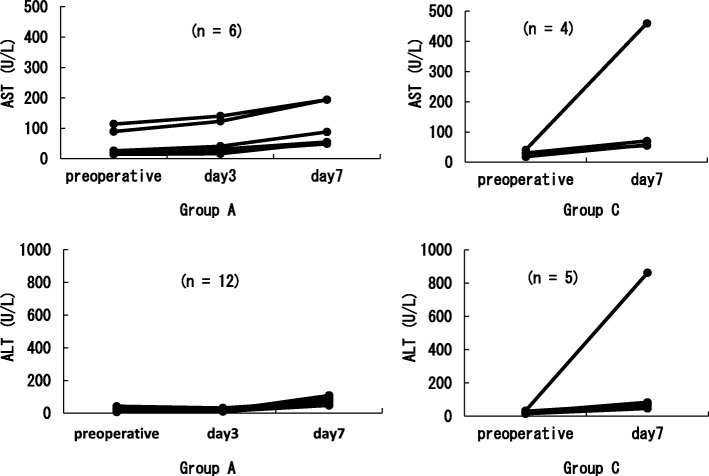
Changes in AST and ALT (more than 1 grade up): Preoperative to Postoperative Day 7. The two upper charts show AST transition and the lower two charts indicate ALT transition.

Figures 2 and 3 show the occurrence of liver dysfunction on day 7 after the surgery. While there was no significant difference between Group C (13.3%) and Group A (21.4%) in the AST level, the ALT level showed a significant increase in Group A (46.4%) compared with that in Group C (16.7%) (*p* = 0.043). In most cases, however, the grade only increased from G-0 to G1. Regarding the over-time trend for changes, all the symptoms including liver dysfunction improved in Group A, and the respective test results returned to their preoperative levels without any treatment. In Group C, except two cases wherein medication treatment was applied for liver dysfunction, all other cases improved without intervention. There was only one liver dysfunction case where both AST and ALT increased to more than the G2 level, and there were two cases in Group A where only AST increased (Fig. 4).

## Discussion

While postoperative pain management is an important measure to facilitate the patient’s improvement after surgery, opioids show a tendency to suppress intestinal peristalsis and promote nausea and vomiting. Therefore, non-opioid analgesics are recommended [5].

Administration of acetaminophen is one of such methods. In a previous study, a scheduled infusion of acetaminophen could reduce the opioid intake while still maintaining the analgesic effect [6]. In this study, we did not change the fentanyl dosage before and after the acetaminophen infusion. We assumed that acetaminophen would provide an additive analgesic effect, reducing the frequency of administration of non-opioid analgesics. Increasing the dose of opioids to alleviate postoperative pain can result in an increase in other discomforts such as nausea and vomiting, as well as in suppressing intestinal peristalsis. By administering a scheduled infusion of acetaminophen, it was possible to alleviate pain without opioid increase. This approach also reduced the time between the need and actual use of analgesics. We confirmed that postoperative pain was indeed reduced in the patients as a result of scheduled acetaminophen infusions.

Wininger and Zafar et al. have reported the effectiveness of postoperative acetaminophen usage [3, 4]. Both studies, however, focused on a relatively low-invasive laparoscopic procedure. This study, on the other hand, focused on postoperative analgesia for both laparoscopic and open colon cancer surgery. We concluded that acetaminophen should be expected to be effective in invasive open surgical cases of colon cancer.

Liver dysfunction is a known side effect of acetaminophen. Thus, this study paid particular attention to changes in AST and ALT. Our data showed no AST increase after a scheduled additional infusion of acetaminophen, but ALT significantly increased. This result was consistent with the data from the study by Watkins et al., where ALT increased in healthy adults who consecutively took acetaminophen (4 g/day) with or without opioid use [7].

While no ALT increase was noted in the blood on the next day after discontinuation of the scheduled acetaminophen infusion, the blood ALT level did increase above the facility standard on day 7 after the surgery. In the study by Watkins et al., ALT continued to increase even at 4 days after discontinuing the infusion and was maintained at a high level for eleven (11) days [7]. The ALT increase caused by acetaminophen may persist even after discontinuation of the drug. We consider that it is necessary to keep monitoring for liver dysfunction on postoperative day 7 using a blood test.

Some studies, on the other hand, have reported contradictory results, with no difference in liver dysfunction after an acetaminophen infusion [6, 8]. These reports, however, evaluated blood test results on days 1 and 3 after surgery [6] and also used G≧3 as a liver dysfunction indicator, based on CTCAE [8]. In this study, we investigated more concrete effects of acetaminophen on ALT. Most of the cases with an ALT increase in this study recovered naturally without administering medications. In addition, ALT only increased to G1, and we concluded that the scheduled infusion of acetaminophen was not dangerous but rather beneficial as a postoperative pain management approach.

A patient in Group C suffered from dizziness and nausea from pentazocine administration, but no patients exhibited adverse reactions (i.e., skin reactions, anaphylactic shock, aspirin asthma, etc.) from acetaminophen administration. Therefore, we conclude that acetaminophen should be selected as one of the first non-opioid analgesics.

### Research limitations

Since this study was retrospective, there was no comprehensive investigation of side effects, except for liver dysfunction and metastasis. In addition, the number of subjects was limited; therefore, we were unable to determine the direct cause for the increases in AST and ALT levels, which were observed in one case in Group C (increased to G3) and in two cases in Group A (AST increase to G2). All of these cases showed metastasis to the liver as well as a preoperative increase in AST and ALT to the G1 level. Future studies can further investigate the course of action in the case when AST and ALT increase to more than G1 levels.

## Conclusions

A periodic infusion of intravenous acetaminophen after colon cancer surgery was found to be an effective method of pain control and alleviation of postoperative discomfort in patients from the day of surgery and to day 2 after surgery. While constant postoperative monitoring of possible liver dysfunction is needed for at least a week, no serious issues should be anticipated if the patient does not already have liver conditions.

## Abbreviations

ALT: Alanine aminotransferase
AST: Aspartate aminotransferase
CTCAE: Common terminology criteria for adverse events
NSAIDs: Non-steroidal anti-inflammatory drug
SPSS: Statistic package for social science
